# Rapid and Accurate Detection of Plant miRNAs by Liquid Northern Hybridization

**DOI:** 10.3390/ijms11093138

**Published:** 2010-09-07

**Authors:** Xiaosu Wang, Yongao Tong, Shenghua Wang

**Affiliations:** Lab of Bio-Resources and Eco-Environment, Ministry of Education, College of Life Science, Sichuan University, Chengdu 610064, China; E-Mails: wangxiaosu521@126.com (X.W.); gainover@126.com (Y.T.)

**Keywords:** miRNA, liquid Northern hybridization, Oryza sativa, small RNA, oligonucleotide probes

## Abstract

Northern blot analysis is a powerful research tool for discovery, validation and expression of genes, and is currently widely used to detect microRNA (miRNA) accumulation. However, the traditional Northern blot procedure, which is based on a support membrane, is overly elaborate and time-consuming, although it is unsurpassed in accuracy for determining the sizes and amounts of multiple small RNAs sharing high sequence identity. Here we present an alternative method derived from plant miRNAs, liquid Northern hybridization, using fluorescently labeled oligonucleotide probes and characterized by simple and specific miRNA determination and quantitation. The entire detection process is completed within a few hours, and multiple miRNAs can be simultaneously detected in a single experiment.

## 1. Introduction

It has been shown that microRNAs (miRNAs), an abundant family of short molecules approximately 21–25 nt in length, universally exist in plants, animals and microbes and regulate gene expression by complementary nucleotide sequence pairing [1]. It has been estimated that approximately one-third of all protein-coding genes are regulated by miRNAs [2]. miRNAs have important roles in a wide range of biological processes, including plant development, signal transduction, protein degradation, stress (biotic and abiotic) responses, and regulation of their own biogenesis [3–8] and they possess great potential for gene control of insects from plants [9]. To date, the biological functions of most miRNAs are unknown, although more than 10,000 miRNAs have been deposited into the miRBase registry (http://www.mirbase.org/cgi-bin/browse.pl) [10].

Once identified, temporal and spatial detection of miRNA expression are critical requirements for understanding the biological functions of miRNAs. To date, traditional Northern blot is the gold standard for the direct study of gene expression at the mRNA level, although sensitive RT-PCR and high-throughput microarray techniques have been developed. Traditional Northern blot has absolute advantages in accurately determining the sizes of small RNAs and simultaneously displaying the sizes and amounts of multiple small RNAs [11]. The traditional Northern blot hybridization, however, has significant limitations: The protocol is very complex and takes more than two days [12] (http://www.utexas.edu/research/sissonlab/protocols/molecularbiology/miRNA_Northern_Blot.pdf;http://web.wi.mit.edu/bartel/pub/protocols/smallRNA_northern.pdf); the traditional Northern blot hybridization including separation of small RNA fragments by denaturing polyacrylamide gel electrophoresis (PAGE); transfer of the separated RNA fragments onto a nylon membrane; and hybridization and detection of the molecules of interest using probes labeled with ^32^P [13,14]. This lengthy process increases the risk of RNA degradation by RNases. The use of hybridization membranes consumes large amounts of RNA samples and reagents but gives low sensitivity, often resulting in a failure to detect low-abundance miRNAs [15,16]. In addition, this type of hybridization, which is based on radioisotope probes and formaldehyde, poses health risks for users as well as risks to the environment [12]. This paper describes a method named liquid northern hybridization, by which miRNAs can be detected simply, quickly, accurately and cost-effectively. Moreover, this method can permit concomitant detection of more than one miRNA at a time if we use multicolor fluorescent probes.

## 2. Results and Discussion

### 2.1. Sensitivity of Hybridization

**Figure 1:**
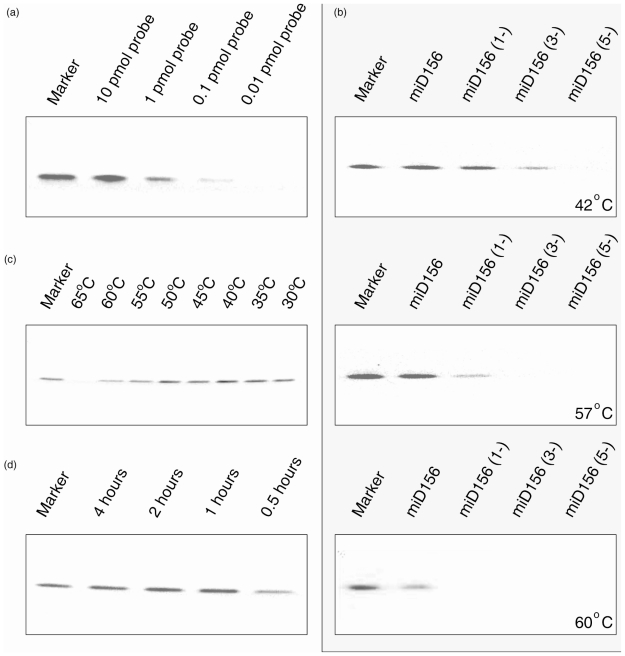
Non-denaturing polyacrylamide gel electrophoresis of liquid Northern hybridization of Osa-miD156* probes with Osa-miD156 (negative image). Hybridization of Osa-miD156* probe with Osa-miR156 as molecular marker. (**a**) Hybridization of 10 pmol of Osa-miD156 with different amounts of Osa-miD156* probes. (**b**) Hybridization of 4 pmol/L of Osa-miD156* probes and 4 pmol/L of Osa-miD156 with different mismatched bases at 42 ºC, 57 ºC and 60 ºC. (**c**) Effects of different temperatures on hybridization of 4 pmol/L Osa-miD156* probes with 5 pmol/L of Osa-miD156. (**d**) The effect of hybridization times on hybridization between 4 pmol/L Osa-miD156* probes and 4 pmol/L Osa-miD156.

We first designed a series of 20-nt oligo-DNAs derived from the rice miR156 sequence (found at http://microrna.sanger.ac.uk) (Table 1) and FITC (fluorescein isothiocyanate)-labeled probe Osa-miD156* ((FITC)-5′-ACGT GCT CAC TCT CTT CTG TCA-3prime;), which contains two additional nucleotides at the 5prime; end [17]. All oligo-DNAs and oligo-RNAs were synthesized by Takara Biotechnology (Dalian) Co., Ltd. Osa-miD156* was hybridized with Osa-miD156 in liquid buffer (buffer 3, see below) at 42 °C for 60 min, followed by digestion of non-hybridized sequences with exonuclease I for 30 min. Non-denaturing PAGE electrophoresis showed that the hybridization signal was visible at FITC-labeled probe levels above 0.1 pmol (Figure 1a). The 0.01 pmol of hybridization signal can be recognized if performing Dot blot (data not shown).

### 2.2. Specificity of Hybridization

To evaluate the specificity of the fluorescently labeled oligonucleotide probe in liquid Northern hybridization, we conducted a series of hybridizations with Osa-miD156* and Osa-miD156s (Table 1) that contain a one-base mismatch, three-base mismatch and five-base mismatch at 42, 57 and 60 ºC, respectively. The results indicated that the degree of tolerance of liquid Northern hybridization to the number of mismatches decreases as the temperature increases (Figure 1b), and only the hybridization signal from the perfect sequence of Osa-miD156 was visible at a hybridization temperature of 60 ºC, which was the melting temperature (Tm) of Osa-miD156* (Figure 1b, lower). This is of special significance, as it distinguishes the member that shares only a one-base difference with others in the miRNA family. However, the fluorescence intensity was stronger when the hybridization was performed 10 °C lower than the Tm (Figure 1c). Moreover, hybridization that proceeded for 0.5 h resulted in a visible fluorescence signal, and a hybridization time of greater than 1 h did not significantly enhance the fluorescence intensity (Figure 1d).

### 2.3. Screening of Hybridization Buffer

**Figure 2:**
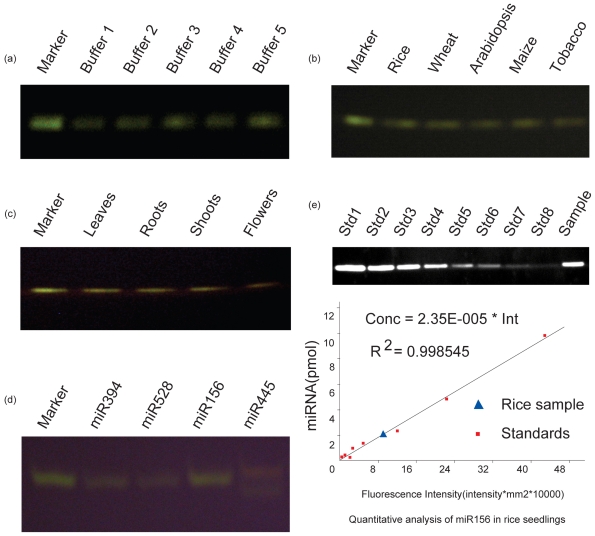
Liquid hybridization detection of miRNAs from different plant materials. Hybridization of Osa-miD156* probe with Osa-miR156 as molecular marker. (**a**) Detection of miR156 from rice seedlings in different hybridization buffers. (**b**) Detection of miR156 from several plant species in buffer 3. (**c**) Detection of miR156 from different rice tissues. (**d**) Detection of different miRNAs from rice seedlings. (**e**) Quantitative analysis of miR156 in rice seedlings by liquid Northern hybridization. Upper: image of miR156 from rice seedlings; lanes 1–8 represent 10 (Std1), 5 (Std2), 2.5 (Std3), 1.25 (Std4), 0.625 (Std5), 0.313 (Std6), 0.156 (Std7) and 0.078 (Std8) pmol, respectively, for creating the standard curve; Lane 9 is a small RNA sample we got from rice seedlings and used 1 ug miRNA (89.69 ng/μL). Lower: image is a quantification using a Bio-Rad gel imaging system.

To screen for the appropriate hybridization buffer, we used small RNA samples from one-week-old rice seedlings to detect the five designed buffers: buffer 1 contained DEPC (diethylpyrocarbonate)- treated water + 10 mmol/L EDTA (ethylenediaminetetraacetic acid); buffer 2 contained 30 mmol/L Sodium phosphate buffer + 10 mmol/L EDTA; buffer 3 contained 0.3 mol/L NaCl + 30 mmol/L phosphate buffer + 10 mmol/L EDTA; buffer 4 contained 2 × SSC + 10 mmol/L EDTA; and buffer 5 contained Northern blotting solution [18]. Small RNAs were extracted from 0.2 g rice seedlings using an isolation buffer according to the method described previously [19], and we used 1ug small RNAs (89.69 ng/μL) in this experiment. The results indicated that the simple salt solution gave satisfactory results for liquid hybridization of miRNA because the hybridization signals were visible in all five buffers, and the signal intensity was not significantly different between buffers 3, 4 and 5 (Figure 2a).

### 2.4. Quantitative Analysis of miRNA

We conducted further quantitative analyses of the electrophoresis results of liquid Northern hybridization of Osa-miR156 from rice seedlings using a Chemi Doc XRS System camera (Bio-Rad, US). A series of hybridizations of different concentrations of Osa-miD156* with synthesized Osa-miR156 was used to create a standard curve. The analysis showed that a good linear relationship exists between fluorescence intensity and miR156 concentration (C = 2.35E-5 * Int. −0.121, R2 = 0.998545), and the expression of Osa-miR156 in rice seedlings is 22 ng/mg fresh weight (Figure 2e).

### 2.5. Simultaneous Detection of Multiple miRNAs

For further verification, we performed an additional detection of miRNAs in 1 μg small RNAs (89.69 ng/μL) from one-week-old rice seedlings using 4 pmol of other miRNA probes (Table 1). The results showed that Osa-miR156 hybridization signals were also detected in different rice organs and other monocotyledonous and dicotyledonous species, for example, *wheat, maize, tobacco and Arabidopsis* (Figure 2b,c), by liquid Northern hybridization. Moreover, the fluorescence signal from the hybridization of Osa-miR156 with Osa-miD156* was brighter than miR394 with Osa-miD394* and miR528 with miD528* (Figure 2d), suggesting that the abundance of miR156 in rice seedlings is higher than that of miR394 or miR528. These experiments have documented that the liquid Northern hybridization technique that we developed is of universal applicability in detecting plant miRNAs. We also added two species of probes, Osa-miD156▴ and Osa-miD445* (24-nt long and labeled with Cy3), into a liquid hybridization system to simultaneously detect their expression levels. Non-denaturing 20% TBE-PAGE indicated that both Osa-miR156 and Osa-miR445 were detected in rice seedlings [20], and the expression of Osa-miR156 (lower in Figure 2d) was less than that of Osa-miR445 (upper in Figure 2d), suggesting that liquid Northern hybridization can provide simultaneous detection of multiple miRNAs in a single experiment.

## 3. Experimental Section

### 3.1. Plant Materials

Seeds of *Oryza sativa L.*, *Zea mays L. and Triticum aestivum L.* were immersed in distilled water for two days and then germinated at room temperature. Two weeks later, seedlings that were approximately 5 cm high were used for RNA extraction. Some seedlings were grown in pots under natural conditions. Leaves, stems, roots and inflorescences or panicles were collected for RNA extraction when the plants reached maturity. *Nicotiana tabacum* plants, the leaves of which were used to isolate RNA, were grown in pots under natural conditions, while *Arabidopsis thaliana (L.) Henyh.(Columbia ecotype)* plants were grown in controlled environment chambers with a 16-h photoperiod at 24 °C. Whole plants were collected for RNA extraction when they grew to the 8-leaf stage.

### 3.2. Extraction of RNA

Total RNA and small RNA were extracted from plant tissues using RNA extraction buffer (1.9 g/L macaloid dissolved in 50 mmol/L Tris-HCl (pH 7.6), 20 mmol/L sodium citrate, pH 7.0, 100 mmol/L NaCl, 10 mmol/L EDTA, 0.5% (w/v) SDS, and 1% (v/v) 2-mercaptoethanol), according to the method described by Chun *et al*. [19] with minor modifications. Extractions of small RNA were performed as follows: Fresh plant tissues (0.2 g) were ground into fine powder with a mortar and pestle in the presence of liquid nitrogen. The powder was immediately placed in an RNA extraction buffer at a ratio of extraction buffer to leaves of 4 mL/1 g and homogenized thoroughly. Then, 1/2 volume of water-saturated phenol and 1/2 volume of chloroform–isoamyl alcohol mixture (24:1, v:v) were added. The contents were mixed well and centrifuged at 12,000 rpm for 10 min at 4 °C. Pphenol/chloroform/isoamyl alcohol extraction was repeated once. The supernatant was mixed with 1/3 volume of 8 mol/L LiCl and incubated at −20 °C for at least 30 min, followed by centrifugation of15,000 g at 2 °C for 10 min. The resulting RNA supernatant was added to 50% PEG 8000 and 5 mol/L NaCl (50 μL of 50% PEG 8000 and 50 μL of 5 mol/L NaCl for every 400 μL of supernatant), mixed and incubated at −20 °C for at least 30 min, followed by centrifugation of 15,000 g at 2 °C for 10 min. The PEG-NaCl precipitation procedure was then repeated. The aqueous phase was transferred to a new 1.5-mL Eppendorf tube, and 1/10 volume of 1 mol/L MgCl_2_ and 2.5 volume of absolute ethanol were added. The mixture was incubated at −20 °C for at least 2 hr, followed by centrifugation of 15,000 g at 2 °C for 30 min. The resulting small RNA pellets were washed with 75% (v/v) ethanol twice, air-dried and dissolved in DEPC-H_2_O and stored at −70 °C.

### 3.3. Synthesis of Oligonucleotides and Hybridization Probes

Five rice miRNA sequences, miR156, miR167, miR394, miR528 and miR445 (Table 1) were found in miRBase (http://microrna.sanger.ac.uk). Their complementary probe sequences, which were labeled with fluorescein FITC or Cy3, and other series of oligonucleotides were synthesized by TaKaRa Biotechnology Co., Ltd., Dalian, China (Table 1).

### 3.4. Liquid Northern Hybridization

A specific amount of oligonucleotide or plant small RNA (1 ug, isolated before) and synthesized probes were added to a 200-μL Eppendorf tube. Hybridization buffer (30 mmol/L Sodium phosphate buffer (pH 8.0), 0.3 mol/L of NaCl, 10 mmol/L of EDTA) was added up to 16 μL. After mixing thoroughly, the reaction mixture was heated to 94 °C for 5 min and incubated in a water bath at 42 °C for 60 min. When the hybridization reaction completed, non-hybridized single-strand sequences, including the hybridization probe, were digested with 10 U Exonuclease I at 37 °C for 30 min. Exonuclease I (NEW ENGLAND BioLabs, M0293) catalyzes the removal of nucleotides from single-stranded DNA in the 3prime;–5prime; direction.

### 3.5. Gel Electrophoresis and Detection of DNA-RNA and DNA-DNA Hybrids

A 0.8-mm thick, non-denaturing 10% TBE-polyacrylamide gel or 10% TAE-polyacrylamide gel was prepared according to Sambrook and Russell [21]. After gel solidification, the above hybridization products were loaded, and the gel was run at 90 V for approximately 40 min. After the run completed, the gel was moved onto a Dark Reader transilluminator (Clare Chemical Research, Inc.) for observation and photography with a Canon A650IS digital camera.

### 3.6. Quantitative Analysis of miRNA in Rice Small RNA Sample

Hybridization of 10, 5, 2.5, 1.25, 0.625, 0.313, 0.156 or 0.078 pmol Osa-miD156* probes with an excess of Osa-miR156 and Osa-miD156* probes were performed with 6 μL small RNA from rice seedlings. After non-denaturing PAGE electrophoresis, the gel was placed on a citaBlue conversion screen in a Chemi Doc XRS System camera (Bio-Rad, US) equipped with a 560DF50nm, 62 mm Emssion filter and auto-exposure. The imaging data were analyzed with Quantity One software.

## 4. Conclusions

These experiments have clearly confirmed that the replacement of solid-phase hybridization by liquid-phase hybridization greatly shortens the time of detection. In addition, the utilization of fluorescently labeled probes instead of radioisotope labeled probes can further shorten the time. Thus, the entire process is simple and easy, and takes only a few hours (approximately three hours in this study) rather than several days as for traditional Northern blot hybridization.

Moreover, for hybridization on support membranes, the effect of hybridization is largely determined by the ability of nucleic acids to attach to the membrane, which is closely related to the sequence length. miRNAs are difficult to firmly absorb on the solid support because their sequences are too short. They are therefore easily washed away from the membrane during or after hybridization. The resulting hybridization signal from some low abundance miRNAs cannot be detected. Such a problem, however, is avoided in liquid Northern hybridization. Hence, through liquid hybridization, it is possible to detect small RNAs that cannot be easily detected by solid-phase membrane hybridization.

Again, some species have very similar sequences in the miRNA family. For instance, in the rice miR160 family osa-miR160e (5prime;-UGC CUG GCUCCC UGU AUG CCG-3prime;) and osa-miR160f (5prime;-UGC CUG GCU CCC UGA AUG CCA-3prime;) have a two-base difference; Another example can also be found in the human miRNA family, where only one-base difference exists between hsa-let-7b (5prime;-UGA GGU AGU AGG UUG UGU GGU U-3prime;) and hsa-let-7c (5prime;-UGA GGU AGU AGG UUG UAU GGU U-3prime;) (http://www.mirbase.org/cgi-bin/browse.pl). Our experiments indicated that liquid hybridization could distinguish one base variation if the hybridization is conducted at Tm (Figure 1b). This will be of particular importance in understanding the functions of miRNA and its regulation of gene expression.

Besides, the method described here can determine the expression level of miRNA in RNA sample through the standard curve method (http://terpconnect.umd.edu/~toh/models/CalibrationCurve.html).

Using fluorescent substances as the markers for hybridization probe has another advantage, that is, simultaneous detection of multiple miRNAs [22]. Different fluorescent materials have different optical properties. For example, FITC shows green fluorescence and Cy3 generates a red fluorescence signal. Multiple miRNAs can be identified in a hybridization reaction if the probes are labeled with multiple fluorophores.

In addition to miRNA, there are large amounts of small RNAs, such as siRNAs, nat-miRNAs (natural antisense miRNA), nat-siRNA (natural antisense siRNA), heterochromatic small RNA and pi-RNAs. They are less than 31 nt in length and broadly present in cells of plant, animal and microbes [23–28], which play important biological functions. This method, developed for miRNAs of plants, can also be used to detect other small RNAs in plants and applied to animals and other multicellular organisms. Hence, liquid Northern hybridization will greatly advance research in the small RNA field. It also opens the possibility of using miRNA expression analysis as a rapid diagnostic tool for plant responses to biotic and abiotic stresses and for human disorders related to miRNAs, such as cancer, immune and nervous system diseases.

## Figures and Tables

**Table 1:** Osa-miRs and derived Oligo-miD and Oligo-miD probe sequences used for liquid Northern hybridization.

Names	Sequence (5prime;–3prime;)	Tm (°C)
Osa-miR156	UGA CAG AAG AGA GUG AGC AC	57.3
Osa-miD156*	(FITC)-A CGT GCT CAC TCT CTT CTG TCA	60.3
Osa-miD156▴	(FITC)-GT GCT CAC TCT CTT CTG TCA	57.3
Osa-miD156	TGA CAG AAG AGA GTG AGC AC	57.3
Osa-miD156 (−)	TGA gAG AAG AGA GTG AGC AC	55.4
Osa-miD156 (3−)	aGA CAG gAG gGA GTG AGC AC	57.4
Osa-miD156 (5−)	aGA CAG gAG tGA GTc AGC gC	59.5
Osa-miR167d	UGA AGC UGC CAG CAU GAU CUG	59.8
Osa-miD167d*	(FITC)-AG CAG ATC ATG CTG GCA GCT TCA	62.3
Osa-miR394	UUG GCA UUC UGU CCA CCU CC	59.4
Osa-miD394*	(FITC)-ATGG AGG TGG ACA GAA TGC CAA	60.3
Osa-miR528	UGG AAG GGG CAU GCA GAG GAG	63.7
Osa-miD528d*	(FITC)-AA CTC CTC TGC ATG CCC CTT CCA	64.0
Osa-miR445a	UAA AUU AGU GUA UAA ACA UCC GAU	52.5
Osa-miR445*	(Cy3)-ATC GGA TGT TTA TAC ACT AAT TTA	52.5

Notes:

* and ▴ represents probe sequence; (−) represents one-base mismatch sequence; (3−) represents three-base mismatch sequence; (5−) represents five-base mismatch sequence. miR156, miR167, miR394 and miR528 contain two additional nucleotides that do not match the target miRNA at the 5prime; end (15).
